# A Dynamical Systems Account of Sensorimotor Contingencies

**DOI:** 10.3389/fpsyg.2013.00285

**Published:** 2013-05-27

**Authors:** Thomas Buhrmann, Ezequiel Alejandro Di Paolo, Xabier Barandiaran

**Affiliations:** ^1^IAS-Research Centre for Life, Mind, and Society, UPV/EHU, University of the Basque Country, San Sebastian, Spain; ^2^Ikerbasque, Basque Foundation for Science, Bilbao, Spain; ^3^Centre for Computational Neuroscience and Robotics, University of Sussex, Brighton, UK; ^4^Department of Philosophy, UPV/EHU, University of the Basque Country, San Sebastian, Spain

**Keywords:** embodied cognition, sensorimotor contingencies, dynamical systems, sensorimotor approach to perception, minimal cognition

## Abstract

According to the sensorimotor approach, perception is a form of embodied know-how, constituted by lawful regularities in the sensorimotor flow or in sensorimotor contingencies (SMCs) in an active and situated agent. Despite the attention that this approach has attracted, there have been few attempts to define its core concepts formally. In this paper, we examine the idea of SMCs and argue that its use involves notions that need to be distinguished. We introduce four distinct kinds of SMCs, which we define operationally. These are the notions of sensorimotor environment (open-loop motor-induced sensory variations), sensorimotor habitat (closed-loop sensorimotor trajectories), sensorimotor coordination (reliable sensorimotor patterns playing a functional role), and sensorimotor strategy (normative organization of sensorimotor coordinations). We make use of a minimal dynamical model of visually guided categorization to test the explanatory value of the different kinds of SMCs. Finally, we discuss the impact of our definitions on the conceptual development and empirical as well as model-based testing of the claims of the sensorimotor approach.

## Introduction

The sensorimotor approach to perception has generated much interest and debate within cognitive science over the last decade. Bringing together insights from various disciplines such as theories of active perception, dynamical systems, ecological psychology, enactivism, phenomenology, cybernetics, and neuroscience, this approach is identified with an influential paper by O’Regan and Noë (2001). In it and in subsequent work, the authors and colleagues have made strong claims about the sensorimotor basis of perceptual experience, namely that both its content and form (what is perceived and how) are constituted by a know-how of sensorimotor regularities or sensorimotor contingencies (SMCs). This perspective rejects the traditional assumption that perception is fully constituted by computations in the brain. Instead, it sees the perceiver as an active agent engaging with the world and perception as intimately linked to skillful action.

In working out the fine-grained details of this idea, different understandings of the sensorimotor proposal have emerged. In general, they vary in how the dependencies are established between both the actual and potential sensorimotor structures that are available to the active agent (and determined by the agent’s situation, bodily skills, and history), and the quality and content of perception. The connection between subpersonal sensorimotor regularities and the personal experience they constitute is made by an appeal to linkage phrases such as “mastery of the laws of SMCs,” “knowledge of SMCs,” or “sensorimotor skill.” For example, O’Regan and Noë argue that in order for a person to be perceptually *aware*, he must not only have acquired mastery of SMCs, but also integrate the current exercise of this mastery with processes of reasoning and action guidance (O’Regan and Noë, 2001, p. 944). Much of how the approach should be interpreted hinges on how such linkage terms are understood and ultimately operationalized.

The details of this passage between the subpersonal and personal levels have been the object of much debate (Hutto, 2005; Clark, 2006; Roberts, 2009). It is unclear whether the sensorimotor approach should be understood as a radical departure from traditional computational functionalism or as an enrichment of it. The debates about how this contentious linkage terminology is best interpreted will probably continue, but in this paper we want to draw attention to an important aspect that seems to have been overlooked in these discussions and in the primary literature: the notion of SMCs itself.

An examination of this notion is a step prior to resolving the central issues in ongoing debates. Far from being a straightforward idea, in this paper we argue that the concept needs a clear definition and admits several refinements. We identify four distinct notions of SMCs: sensorimotor environment, sensorimotor habitat, sensorimotor coordination, and sensorimotor strategies. We define these concepts operationally, thus filling what we see as a gap in sensorimotor theory. The four sensorimotor structures are described using dynamical systems terminology although we also show that they can be investigated by a variety of methods. We do not claim that other useful operational definitions are not possible, but we think our definitions cover most of the usages we have identified as intended explicitly or implicitly in the sensorimotor approach. Then we evaluate the usefulness of these concepts by applying them to the analysis of a new minimal cognition model of active categorical perception (following work by Beer and others, e.g., Beer, 2003). This achieves the double purpose of testing the operational character of the proposed ideas and of gaining further insight into their relation. Finally, we discuss the theoretical, empirical, and modeling impact of these four kinds of SMCs.

## What Exactly is a Sensorimotor Contingency?

The idea that sensorimotor regularities play important roles in perception is not new. Inspired in the work of MacKay (1962), the notion of SMCs is closely related to concepts such as sensorimotor invariants, sensorimotor correlations, and sensorimotor loops. Among the approaches that directly build upon sensorimotor dynamics we find: ecological psychology (Gibson, 1979; Warren, 2006; Turvey, 2007), adaptive behavior dynamical modeling (Ashby, 1952; Powers, 1973; Beer, 1990, 1997), autonomous robotics (Walter, 1953; Braitenberg, 1986; Brooks, 1991) enactive cognitive science (Varela et al., 1991; Thompson, 2007; Di Paolo et al., 2010; Stewart et al., 2011), synergetics, and coordination dynamics (Kelso, 1995, 2009), sensorimotor constructivism (Piaget, 1947, 1955), embodied accounts of infant development (Thelen et al., 1994), experimental work on sensorimotor disruptions (Kohler, 1962, 1964; Taylor, 1962), and sensory substitution (Bach-y-Rita, 1972; Lenay et al., 1997; Auvray et al., 2007; Froese et al., 2012), and even some forms of “good old fashion” behaviorism (Hull, 1937, 1943). Philosophical precursors of SMCs include Dewey’s (1896) critique on the notion of reflex arc, Husserl’s (1997) phenomenological insights on spatiality, and Merleau-Ponty’s (1945/2012) concept of motor intentionality.

There have been very few attempts to define SMCs formally, a required step for testing the claims of the sensorimotor approach by means of modeling and empirical work. This situation is confirmed by the paucity of models examining the main tenets of the theory. Some of the few existing models are rather abstract and focus almost exclusively on the problem of extracting the proper dimensionality of the interaction space of an agent (Philipona et al., 2003; Philipona and O’Regan, 2010). Others focus more directly on robotic applications and assume a probabilistic, discrete-time interpretation of SMCs (Maye and Engel, 2011).

The concept of SMCs seems to point in an unproblematic manner to regularities in the sensorimotor field: predictable or “lawful” co-variations of sensory stimulation, neural, and motor activity. For instance, the projection of a horizontal line onto the retina changes from a straight line to a curved arc as one shifts the eye’s fixation point from the line itself to points above or below it. In contrast, if the focus is moved along the line no such transformation takes place. The geometry of the viewed object, the morphology of the retina, and the particular movement pattern employed, all determine regularities in sensory stimulation (O’Regan and Noë, 2001, p. 941). Such regularities could in principle be described if enough detail is known about the sensory system and environment.

However, what counts as a sensorimotor dependence can change if we decide to focus on all possible scenarios given the details of the agent’s sensory and motor systems and its surroundings, or if we study the agent as the partial creator of such regularities, or if we consider different task-oriented scenarios with different patterns of saliency. The idea of SMCs also suggests the relevance of regular structures in these dependencies, but what counts as regularity can depend on the scale of observation, on whether we make purely dynamical considerations or whether the focus is on the functional organization of the task, and so on.

O’Regan and Noë’s original formulation describes SMCs as follows:

“[…] the *structure of the rules* governing the sensory changes produced by various motor actions, that is, what we call the *SMCs* …” (O’Regan and Noë, 2001, p. 941; emphasis in the original).

The original paper, and the ones that followed it, do not offer more explicit definitions. We can, however, illustrate the way in which the concept has been used:

“According to this theory, seeing is a skillful activity whereby one explores the world, drawing on one’s mastery of the relevant laws of sensorimotor contingency” (O’Regan and Noë, 2001, p. 966).

“Now, perceiving, according to the sensorimotor contingency theory, is an organism’s exploration of the environment that is mediated by knowledge of SMCs” (Myin and O’Regan, 2002, pp. 33–34).

“(…) to see something is to interact with it in a way governed by the dynamic patterns of sensorimotor contingency characteristic of vision, while to hear something is to interact with it in a different way, governed by the different patterns of sensorimotor contingency characteristic of audition” (Hurley and Noë, 2003, p. 146).

As we have said above, the precise nature of linkage terms like “mediation,” “mastery,” etc. is unclear and can generate practical uncertainty at the time of designing an experiment or modeling the behavior of a robot. But we also believe that all of this uncertainty relates to the lack of specificity of the SMCs concept itself.

For instance, the majority of examples of SMCs employed in the literature, such as the one involving the change in curvature of a line projected onto the retina as the eye moves, or the lawful change in the properties of light reflected from an object’s surface as the observer, the object or the light source move around (O’Regan and Noë, 2001, p. 942; Philipona and O’Regan, 2006), or the set of distortions that a shape undergoes as its position relative to the observer changes (O’Regan and Noë, 2001, p. 942), all have in common that they describe the static sensory consequences of arbitrary motor changes, i.e., the immediate, local effect of an agent’s change from one state to another. Other examples, in contrast, acknowledge the role of the agent’s driven, ongoing engagement with the environment in the creation of invariant sensorimotor structures. For example, O’Regan and Noë (2001, p. 956) refer to sensory substitution experiments by Lenay et al. (1997), in which the distance of a light source can be estimated using only the vibration created in response to a single on-off photo-sensor fixed to the hand. This is possible only when subjects perform movements along an arc, such that elbow and shoulder angles covary according to a fixed relation, while the light remains fixated. Here, the relevant contingency is not found in the instantaneous consequences of arbitrary observer motion, but is rather created through a sensorimotor engagement that couples specific movement patterns with sensory feedback. While such examples hint at different possible interpretations of the notion of sensorimotor contingency, such differences have so far not been made explicit in the literature.

The usage of the term SMC suggests that there might in fact be not one, but a range of useful interpretations. SMCs can refer to the lawful relations between all possible sensory and motor states, given an environment and a sensorimotor apparatus, but independently of what the agent is actually doing. It could otherwise point to a set of specific dynamical structures (e.g., attractors, metastable regions) in the space describing sensorimotor configurations. Alternatively, they could indicate the correlations and sensorimotor co-variations that arise from the agent’s actual behavior; and the latter might even break up into different concepts depending on the level of skill deployed by the agent and the timescale of interest.

These different forms of dependency on the agent’s body, internal dynamics, overall cognitive strategy, and skills call for a disambiguation of the concept of SMCs into distinct workable notions and an examination of the relations they bear among each other. These different ideas cover at one end broad descriptions of all the possible lawful structures that can be identified in an agent-environment interaction and, at the other, specific forms of sensorimotor coordination that are strategically deployed by the agent in a goal-oriented or normative manner.

## Four Kinds of SMCs

We can distinguish sensorimotor relations in senses that vary with the degree of agent-centredness. One possible relation describes how sensory input changes with induced motor activity in an open-loop fashion. This depends on the embodiment of the agent and the environment only, not on what the agent is actually doing. Another relation looks at co-variations that obtain once the loop is closed by taking into account the agent’s internal activity and responsiveness to sensory changes. The next sensorimotor relation is more specific and looks at the coordination patterns that contribute to the performance of a task. And finally, another sensorimotor relation indicates how such coordination patterns may be organized normatively so as to distinguish levels of skilfulness, efficiency, stability, etc.

**Figure 1:**
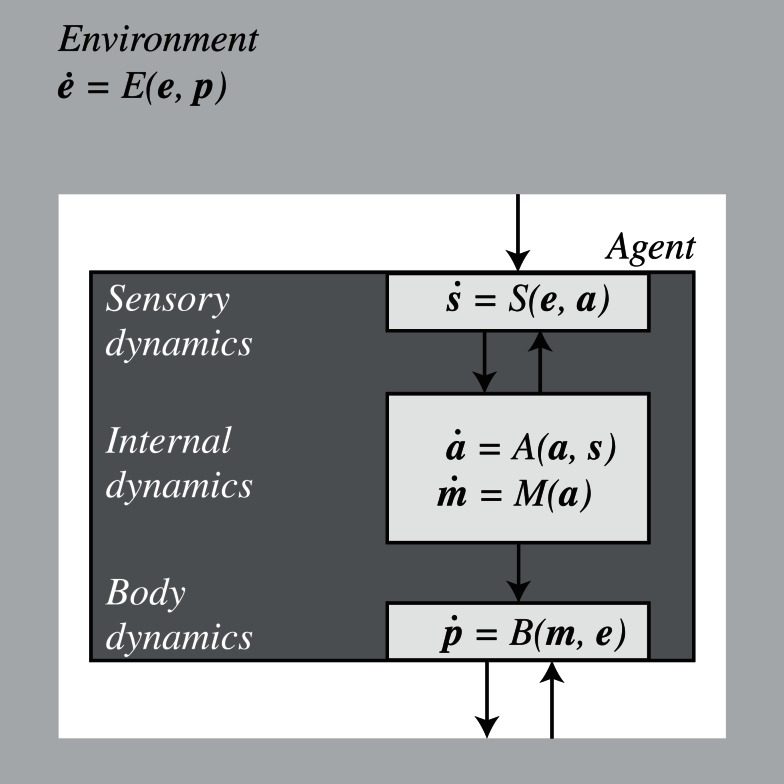
**Agent and environment as coupled dynamical systems described by differential equations**. The world is partitioned into components describing the dynamics of the environment (**e**), the agent (in bodily configuration **p**), and their interaction. The agent is further subdivided into sensory (**s**) and motor interfaces (**m**) as well as its internal state (**a**). For further details see text.

One-way to shape these intuitions is to formalize the sensorimotor coupling of an agent with the environment using a dynamical systems approach. In general, such a system could be described by the set of equations shown in Figure 1.

The details of these equations depend on the specific model under consideration and the level of relevant detail. The important point is that for every agent-environment system we can choose some variables as describing the environment and some as describing the agent. For our purposes, the choice is conventional and the distinctions could be drawn differently depending on the focus of interest. Here we do not discuss how this and other distinctions could be drawn as a matter of principle, but accept that in specific application scenarios they are often unproblematic. In some cases we may not wish to make explicit, say, sensory activity as a variable of interest but would be happy to describe the agent as a whole coupled with the environment as a whole (with sensor inputs as parameters as in, e.g., Beer, 2003). In other cases, depending on the interest, we may wish to distinguish, as we do in this paper, between “bodily” variables, including anatomical structures, posture, sensors, and effectors, and “internal” variables such as those that could represent neural activity (which arguably are also part of the body), and so on. It is important that once a distinction has been drawn, whether by convenience or as a matter of principle, this distinction should be maintained. As long as this rule is observed, the following analysis will remain valid.

With these caveats in mind, we describe the environment by a function *E* that assigns changes in the value of an environmental state **e** to each agent’s body position/configuration **p** in the world and takes account also of its own independent dynamics. The position vector, **p**, describes in general the whole body configuration of the agent in relation to its environment. Sensors (*S*) transform environmental states **e** (which depend on **p**), into sensory states **s**, which in turn modulate the agent’s internal state **a**. Motor commands **m** [i.e., the outflowing (efferent) movement-producing signals closest to the periphery], which are a function of the internal state, activate effectors in the agent’s body *B* that lead to changes in body configuration **p**, thereby closing the loop. The sensor states, **s**, will normally also be dependent on internal factors (**a**) such as neural dynamics. They will also depend on body configuration (**p**) although this is already factored in the environmental state **e** that depends on **p** – this includes dependencies due to displacements of the body as well as active changes introduced in the environment by the agent^1^.

Using this abstract formalization of the sensorimotor coupling we identify four notions of SMCs.

### Sensorimotor environment

As pointed out above, most examples of SMCs in the literature refer to the instantaneous sensory consequences of arbitrary changes in perspective or movements in general, without considering how the movements themselves are related to sensory feedback (i.e., regarding these changes from an open-loop perspective). Movement of the body leads, for example, to lawful instantaneous changes in the optic flow on the retina (expanding when moving forward, contracting when moving backwards, etc.). Equally, rotations of the head lead to a lawful change in the temporal asynchrony between sounds received in the left and right ear (O’Regan and Noë, 2001, p. 941). These lawful relations are independent of how such movements originate.

We can capture this kind of SMCs by considering how an agent’s sensor values **s** change in relation to given motor states assuming that the motor command **m** varies freely – in other words, **m** is taken as an independent variable, decoupled from the agent’s internal variables **a**. The sensorimotor loop is opened by removing the equation **ṁ** = *M*(**a**). This relation can depend on several environmental and bodily factors – for instance, the position/configuration **p**, considered a parameter in this open-loop version of the model. In the most general terms, this is expressed as an implicit function involving sensor and motor values: *f* (**s**, **m**) = 0, where **m** is the independent variable. In some cases, this relation can be expressed explicitly as **s** = *g*(**m**). Mathematically, relevant aspects of this relationship could be captured, whenever possible and at least locally, by the partial derivative ∂**s**/∂**m**, i.e., the change in sensor values **s** resulting from changes in the independent variable **m** while all other variables are held constant.

We call this functional relation (*f* or *g*) the *Sensorimotor Environment*, which is specific to agents that share the same sensorimotor embodiment and external environment, but is independent of the agent’s internal state and the actual actions it performs. The SM environment constitutes the set of all possible sensory dependencies on motor states (**s**, **m**) for a particular type of agent and environment. Whatever specific behavior the agent exhibits, its sensorimotor projections will always be found within this set, which constitutes the most abstract sense of sensorimotor dependencies (and the type most often illustrated in the literature).

Identifying the SM environment can be very useful, as we shall see in the next section. This set will have several properties of smoothness, dimensionality, and symmetry that constitute the most general constraints to any actual sensorimotor trajectory for a given agent, including both successful and unsuccessful strategies for solving a given task. The properties of the SM environment are the most general kind of regularities or “laws” of SMCs. They are shared by all agents with the same bodies in a given environment.

### Sensorimotor habitat

In contrast to regularities that can be identified in the SM environment, some SMCs are created only through specific patterns of agent-environment interactions, i.e., in the time-extended closed-loop coupling between motor variations and sensory feedback. Such is the case, for instance, in the example of minimal sensory substitution described above, in which the distance of a light source can be determined only by performing a certain class of arm movements while keeping the light source fixated (Lenay et al., 1997). Other examples include perceiving the softness of a sponge (which we will come back to below), or the strategies employed by baseball outfielders, who move in such a way as to create and exploit certain sensorimotor invariants in order to catch fly balls without having to predict where and when the ball will land (Chapman, 1968; Sugar et al., 2006).

In our formal framework we identify SMCs of this kind with regularities in how an agent actually “navigates” the SM environment. We close the loop again and take into account the agent’s internal state and its influence on the effectors. We define the *Sensorimotor Habitat* as the set of all sensorimotor trajectories that can be generated by the closed-loop system, i.e., taking into account the evolution of the internal states **a**. This structure describes how an agent “moves” within the SM environment, the different instantaneous tendencies, the regions that are most likely to be visited, the temporal patterns of these trajectories, and other regularities. The SM habitat inherits some constraints from the SM environment but it is likely to be of a higher dimensionality because of the addition of internal dynamics (and consequently the temporal dimension). In other words, although the SM environment constrains the possible habitats, there are still an infinite number of ways in which the SM environment can be “inhabited.”

Formally, the SM habitat corresponds to the set of actual sensorimotor trajectories traveled by the closed-loop system (equations in Figure 1) for a range of values of relevant parameters (initial positions, initial states, environmental parameters, etc.). Whenever possible, local information about the SM habitat can be captured by the total derivative d**s**/d**m**, using the full set of equations, where **m** is not any more a free variable but is constrained by the agent’s internal states, **a**, and, needless to say, the coupling of the agent with its environment. As is the case with the SM environment, the SM habitat will have certain properties of smoothness, dimensionality, topology, and symmetries to which we can now add dynamic attractors that characterize a particular type of agent and its environment. These regularities specify SMCs that take into account the active engagement of agent and environment.

It is important to note that neither the SM habitat, nor its properties, can in principle be fully deduced from the SM environment. It is true that whatever the SM habitat, its existence must be a possibility in the SM environment. And in specific cases properties of the former might reflect properties of the latter. But the agent’s internal dynamics also create completely new behavioral constraints and new ways in which sensors and motor can be coupled in a time-extended manner. A metaphor might be to think of the SM environment as the room within which a person is doing some activity. The walls of the room constrain the possible behaviors but cannot determine whether the person will sit in meditation, lie down, or do exercise, activities that would be described by the SM habitat. In other words, the “laws” of the SM environment constrain but do not fully determine the regularities of the SM habitat.

### Sensorimotor coordination

So far, we have considered the agent outside of any functional context (task performance, maintaining viability, etc.). Within the SM habitat we may find certain regularities as we have said. Some dynamical patterns may be repeated for a large set of parameter values, there may be (meta)stable trajectories, and even transients may occur reliably for a set of circumstances, things we would describe as what an agent normally does. Some such regular patterns are often found to be crucial for task performance in the area of autonomous robotics (Pfeifer and Scheier, 1997; Beer, 2003). We call any such reliable or (meta)stable pattern a *Sensorimotor Coordination* if it contributes functionally to the performance or goals of the agent.

For example, the presence or absence, depending on the environment, of a stable oscillatory attractor within the SM habitat could lead the agent to perform a categorical discrimination (see Beer, 2003, and the example in the next section). Or a correlation between self-sensed left and right angular velocities of the wheels in a robot while keeping a proximity sensor activity nearly constant could be used to discriminate between cylinders of a large or small diameter by indirectly “measuring” their size (Pfeifer and Scheier, 1997). Or in the now classical example, the softness of a sponge can be determined by squeezing it between the fingers (Myin, 2003), the quality of interest resulting from a specific correlation between felt pressure and resistance. All these are cases of SM coordination, i.e., SMCs described by co-dependencies between **s** and **m** that reliably contribute to functionality.

SM coordination patterns are characterized by a significantly lower dimensionality than the SM habitat as a whole – they are specific, often local sensorimotor co-dependencies, SMCs, that are dynamically organized in time – but they are not necessarily always (meta)stable. Even transients, as long as they are “used” reliably, could be explanatorily linked to functionality and so count as SM coordinations (see next section).

In practical terms, a SM coordination pattern is determined by a dynamical analysis of the agent within the context of a given task or performance. It will inherit dynamical constraints from the SM environment and the SM habitat and in general some of the most likely candidates will come from regularities in the SM habitat. However, not all regular patterns in the SM habitat will necessarily be SM coordinations, as some of them may have no functional significance.

This qualification allows us to bring some clarity to O’Regan and Noë’s example of the missile guidance system, of which they say that it is “‘tuned to’ the SMCs that govern airplane tracking,” while an out-of-order missile guidance system is said to only have “a kind of ineffectual mastery of its SMCs” (O’Regan and Noë, 2001, p. 943). Within our proposed framework we can avoid referencing seemingly paradoxical concepts such as “ineffectual mastery.” A correctly functioning missile, which fulfils the goal of tracking airplanes, can simply be said to exhibit SM coordinations. An out-of-order missile, in contrast, might still perform stable trajectories, but if these are not efficacious for the tracking of airplanes, we do not consider them SM coordinations.

### Sensorimotor strategies

Until now we have described different sensorimotor structures in terms of their dynamical properties and their functional contribution, but without reference to other normative or adaptive dimensions, i.e., according to measures of efficiency or level of skill. The explanatory value of the sensorimotor approach is, however, often expressed in normative terms such as “being attuned to SMCs,” possessing “knowledge,” or “skillful mastery” of the laws of SMCs. Consider for example the discussion by O’Regan and Noë, 2001, p. 942) about congenitally blind people who recover vision after undergoing a cataract operation. These patients must learn from scratch different visual attributes (e.g., the changing shapes of a disk that depend on the subtended angle, which in the case of normal vision are nevertheless perceived as always belonging to a disk). Presumably, the lawful covariation of shapes with respect to eye movements is the same in the case of these patients as in people with normal vision, i.e., they all share a similar SM environment. And after some basic training they would also share a similar structure for the SM habitat and similar patterns of SM coordination as they learn visual tasks. And yet, for normally sighted people, visual attributes are deeply ingrained and their perception is already adjusted to a level of visual performance required by constraints of speed, efficiency, etc., while for people with recovered vision, the task of learning them to the point they must not think explicitly about them takes quite some time. This difference motivates the proposal of a sensorimotor concept able to account for variations in the larger organization of SM coordination patterns according not yet to a level of mastery or lack thereof, but more neutrally, to norms in general.

A normative framework involves reference to given criteria that distinguish or value some possible outcomes above others: a dexterous movement vs. a clumsy disaster, achieved know-how vs. lack of experience, and so on. We are not going to discuss the origins of such norms here, we will just assume that they exist and that one can provide some kind of normative gradation, such as efficiency, fitness, optimality, or even subjective criteria like hedonic value.

Performing a complex task requires various SM coordination patterns spread out in time, sometimes in sequential order, sometimes in parallel, and involving different action and perceptual systems. Even if several such combinations may be efficacious, some may be more efficient than others. Within a normative framework we can introduce the notion of a *Sensorimotor Strategy*. The idea describes an organization of SM coordination patterns that is regularly used by the agent because it has been evaluated as preferable (along some relevant normative framework) for achieving a particular goal. The development or acquisition of a SM strategy describes how an agent becomes attuned to a specific situation, by selecting and modulating SM coordination patterns in accordance with relevant norms. The notion corresponds to an even more agent-centered and history-dependent idea of SMCs and it seems close to some of the uses of the term that link SMCs and personal level phenomena.

A discussion of possible interpretations of notions such as “task,” “goal,” or of the origin of norms is outside the scope and not the purpose of this paper. Suffice it to say that it does not matter if one chooses to ground norms in cybernetic ideas about mechanisms for flexible and robust achievement of final conditions (Rosenblueth et al., 1943); in evolutionary considerations of how a trait has contributed to a species’ survival (Millikan, 1989); or in enactive concepts of agency and sense-making (Di Paolo, 2005; Di Paolo et al., 2010), to name but a few. Our distinction of kinds of SMCs is independent of these considerations and can be applied once one has settled on a suitable understanding of the terms in the context of a particular problem case.

The above definitions are operational. However, in practical cases it is rare to be in possession of all the relevant dynamical equations. This does not preclude the use of other methods, such as probabilistic and information-theoretic analyses (see Discussion) for studying the regularities of the different kinds of SMCs.

Table 1 summarizes the four kinds of SMCs.

**Table 1:** **Summary of sensorimotor structures (SMCs) indicating dependencies on different factors**.

	Environment	Embodiment	Internal activity	Task	Normative framework
**SM ENVIRONMENT**					
The set of sensory states as a function of motor variations independently of the agent’s internal (e.g., neural) dynamics	✓	✓			
**SM HABITAT**					
The set of possible sensorimotor trajectories traveled by a closed-loop agent for a range of values of relevant parameters	✓	✓	✓		
**SM COORDINATION**					
Individual trajectories within the SM habitat that occur reliably and contribute functionally to a goal	✓	✓	✓	✓	
**SM STRATEGY**					
Efficient organizations of SM coordinations developed, acquired or selected as a consequence of being normatively evaluated	✓	✓	✓	✓	✓

### An example

Let us consider how these notions can be applied to Myin’s (2003) example of perceiving the softness of the sponge by squeezing it between the fingers. For simplicity we assume that a sponge is being held between thumb and forefinger, and that the motion is constrained to pinching movements. The relevant sensory variables include the pressure felt on the surface of the skin, proprioception, and the effort required to maintain a certain grip.

The SM environment describes the sensory consequences of all possible motor commands. It expresses, for example, how tactile sensation in each finger is related to pinching movements, and identifies the positive correlation between sensed pressure and the closing of the grip. It also captures the fact that tactile sensation vanishes if the distance between the fingertips is larger than the sponge, or that greater muscle effort is required to maintain finger positions as one brings the fingertips together.

The SM habitat can be described only if we know-how the person will respond to the various sensations, i.e., if we close the loop. We could assume, for example, that the subject has the tendency to squeeze the sponge until a given level of resistance is met, at which point the direction of movement is reversed until a minimum pressure threshold is reached, and the squeezing re-starts. The SM habitat, in this case, exhibits regularities that reflect the resulting rhythmic pattern and the points of movement reversal. Note that these features cannot be deduced from the SM environment, and that the SM habitat in this case includes not only sensorimotor variables, but also internal variables that play a role in the generation of the particular behavioral pattern.

To speak of SM coordination we need to link behavior to a task. Let us assume that the person wants to discriminate between soft and hard sponges. One solution to this task relies on the rhythmic pattern just described. Proprioceptive feedback at the high resistance turning point of the movement indicates how resistant the sponge is in term of spatial displacement. Small displacements mean the sponge is hard, and it is softer in the case of larger displacements. Alternatively, a similar coordination could control the pressing movement so as to always obtain the same displacement. In this case the pressure sensation would be indicative of the degree of softness. Both patterns constitute SM coordinations because they are efficacious for the distinction of soft and hard sponges. Other types of movements, such as moving the fingers along the surface of the sponge with minimal pressure, would not have this property.

The SM coordination patterns described can be enacted in a variety of ways, all of which will solve the task. Repeated squeezing, for example, might be helpful in getting a more reliable estimate of sponginess. How to approach these choices will depend on a normative framework, e.g., whether the subject cares about speed, accuracy, efficiency, and so on (imagine the choices faced by a worker whose daily task is to sort out thousands of sponges into soft and hard). The way these options are structured determine the SM strategy.

The example is a good indicator of the insights that may be gained from unpacking the idea of SMCs into four sensorimotor concepts. The dependence of the perception of softness on the act of squeezing the sponge is a good illustration of a strong relation between perceptual experience and action. And yet to propose that this relation is one of mastery of sensorimotor laws is too coarse a statement because, as the example shows, it is not simply a question of the agent being sensitive to how sensation varies with movement in general (the SM environment). What matters in this case is also how these structures can be used to regulate an active movement pattern (SM habitat). This pattern itself gives rise to novel regularities, which are possible, but not pre-determined by the laws of the SM environment, and these regularities can then be put to the task of discriminating actively according to perceived softness (SM coordination) and, further on the scale of mastery, according to other norms of interest (SM strategies). A correct, but all too broad declaration of dependence of perception on action has now been unpacked into the finer-grained analysis afforded by the new distinctions.

## A Minimal Model: Categorical Perception

In this section we use a minimal model to illustrate the different kinds of SMCs proposed and identify the roles they play in the behavior of an agent engaged in a task. The purpose is to demonstrate how the concepts can be applied as well as to generate new insight into their relations. The model is deliberately simple but not trivial, following the tradition of minimal cognition models introduced by Beer (1997, 2003) and others (Cliff, 1991; Harvey et al., 1996). Specifically, we consider a minimal model of active categorical perception that allows us to illustrate and analyze in detail the agent’s sensorimotor dynamics. We keep technical details to a minimum. More information can be found in the Appendix.

**Figure 2:**
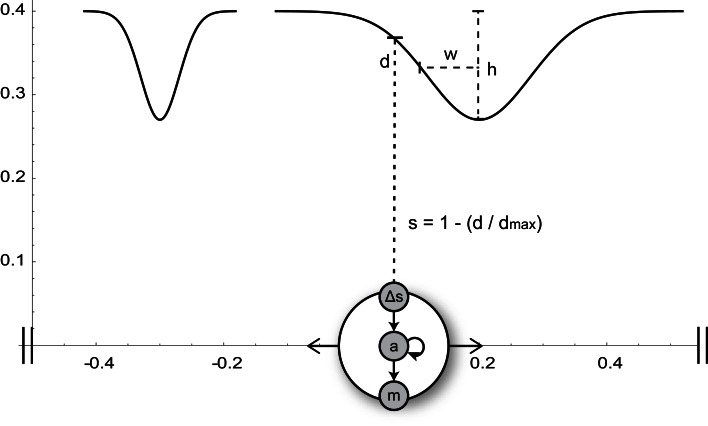
**Minimal agent and its circular 1-D environment (horizontal axis)**. The agent, represented by a big circle, can sense the normalized proximity s to objects in front of it (narrow or wide Gaussian shapes of height h and width w). The time-derivative (Δs) of the sensor signal serves as input to an interneuron (a) of the agent’s neural network. The interneuron is recurrently connected to itself and drives the motor neuron m, which controls the agent’s horizontal velocity.

In our model, an agent can move along a one-dimensional environment that contains visual stimuli in the form of two bell-shaped gradients of different widths (Figure 2). The agent can sense these shapes via a single distance sensor. The activity of this sensor increases proportionally to the proximity of the object directly in front of it. The time-derivative of the sensor signal serves as input to a small dynamic neural network that delivers continuous motor commands controlling the agent’s velocity.

The task to be solved by the agent is the discrimination between wide and narrow shapes, requiring it to move away from the former, and approach the peak of the latter (this is a continuous version of the discrete robot task in Maye and Engel, 2011). The situation is somewhat similar to a blind person exploring different shapes with the help of a cane only. No instantaneous sensory input can bear enough information to discriminate one shape from the other (the height and horizontal positions of the two shapes are varied such that within each trial they can be distinguished only by their steepness and horizontal extent). Consequently, an active sensorimotor strategy is needed. The parameters of the neural network are artificially evolved such that the agent is able to solve the discrimination task, and the best solution is analyzed in some detail in the rest of this section.

### Results

The best evolved agent behaviorally distinguishes shapes across a range of widths and heights (exhibiting generalization with clear categorical boundaries, see Appendix). In the case of narrow shapes the agent approaches at constant velocity and starts oscillating once in contact with the shape. The pattern is similar to those found in analogous sensory substitution devices (Froese et al., 2012). This oscillation is asymmetric, and such that the agent slowly approaches the peak, close to which it ultimately settles with decreased oscillation amplitude. For wider shapes, the initial approach is identical. But instead of moving in a cyclical fashion toward the peak, the agent moves away from it.

In what follows we explain how the approach and avoidance behaviors result from the agent’s sensorimotor coupling with the environment as described by the different sensorimotor structures proposed above.

#### Sensorimotor environment

**Figure 3:**
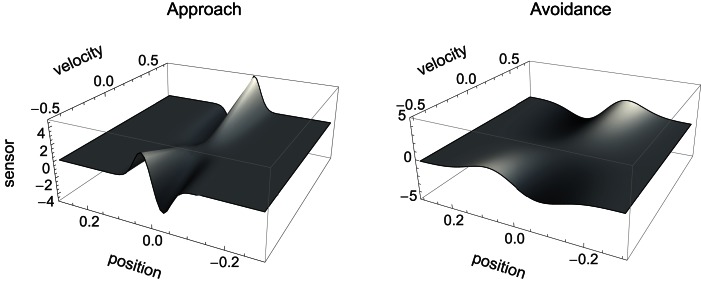
**SM environment for approach and avoidance conditions: sensory change that results from issuing a motor command that determines agent velocity at a given position**. Slices of this surface along the position dimension correspond to derivatives of the original Gaussian shape (which is not surprising as the sensor outputs the time-derivative of measures distance). The amplitude of this derivative depends on the agent’s velocity.

The sensorimotor environment, to recapitulate, captures properties of the external environment and the agent’s sensorimotor interface, without taking into account the agent’s internal dynamics. In Figure 3, we have plotted the change in sensory values that results from the agent being located at a given position relative to the center of a shape and having issued a certain motor command (moving at a certain speed). Since the agent senses the time-derivative of the height of the gradient at its current position, a specific change in sensor value is produced for each position and velocity of the agent. The resulting surfaces shown in Figure 3 (narrow shapes on the left, wide shapes on the right) represent the agent’s SM environment in that they capture the functional relation between **s** and **m**, i.e., the sensory consequence of performing an action (moving at velocity *v*) taking position p as a parameter.

Although the two SM environments present a similar shape, the shallower gradient (on the right of Figure 3) leads to lower absolute sensor values. What are the regularities of this surface (its “laws” of SMCs), and how do they contribute to explaining the agent’s behavior? It is clear that there are two types of symmetries in the sensorimotor environment: those reflecting properties of the external environment, i.e., the bell shapes, and those reflecting the agent’s motor abilities, i.e., the fact that the agent can move equally both to the left and to the right. As a result, we notice that sensor values as a function of position and velocity observe the symmetry *S*(p, *v*) = *S*(−p, −*v*). Specifically, the sensorimotor surface features two symmetrically arranged peaks and troughs that correspond to the points of greatest slope of the bell shape. These peaks are found at either side of the bell curve (leading to the first symmetry) and change in sign if the agent’s velocity changes direction (leading to the second symmetry). The surface also reflects other general properties of the sensorimotor coupling, such as the fact that sensor activity is continuous and smooth.

The structure of the SM environment, in this case for example its symmetry, can constrain but does not fully determine the possible behavioral strategies for solving the task. For example, without any further knowledge of the agent’s internal structure, the SM environment predicts – and this has been confirmed – that if the sign of the motor signal was inverted (effectively exchanging the left/right directions), then the same discrimination behavior would be observed, but with the difference that the agent would now “scan” the shapes on the opposite side of the peak.

**Figure 4:**
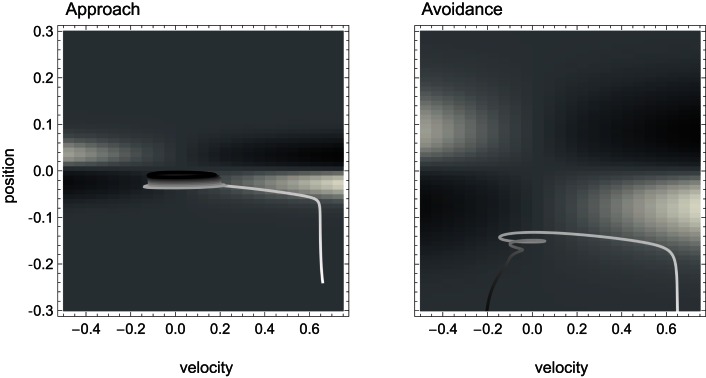
**Agent trajectories within the sensorimotor environment for approach and avoidance conditions**. The SM environment is plotted here as a “top-view” of Figure 3. On a background of gray areas indicating no sensory change, brighter points correspond to more positive and darker points to more negative change in sensory value. The shade of the agent’s actual trajectory changes from white to black as time progresses. While one can observe oscillations between areas of positive and negative sensory change on the left, and the lack of this pattern on the right, the reason for this behavior cannot be deduced from the SM environment.

The SM environment on its own cannot explain how the agent achieves the distinction between shallow and steep gradients. If we look at the agent’s actual trajectories in the two sensorimotor environments (Figure 4), we can at best come up with heuristic descriptions of the agent’s behavior that seem to involve different types of oscillation between negative and positive sensory regions. But there is no obvious interpretation at the level of the SM environment of why the agent follows these particular trajectories.

For a complete description we need to know how the observed structures of the SM environment (the symmetries in peaks and troughs) affect actual behavior.

#### Sensorimotor habitat

The SM habitat, according to our definition, describes the relationship between sensor activity and motor commands taking into account the internal dynamics of the agent. It is the set of all possible trajectories that the agent takes given a range of boundary conditions and parameters. If we are dealing with a potentially complex, non-linear dynamical agent-environment system, providing a full analytical description of this set may be infeasible. A typical approach is to adopt a quasi-static method. The idea here is to treat the variable that links two components of a complex dynamical system, such as the sensor variable in our model, as a fixed parameter. This removes temporal variation from the dynamical component of interest, and allows one to calculate its qualitative behavior (limits sets, attractor basins, etc.) for the given, now fixed, parameter. In a next step one can then study how the qualitative behavior changes as the parameter is varied (bifurcation analysis). Together, these two analyses approximately describe how the overall behavior of the component results from the change in its qualitative dynamics as the normally time-varying input changes.

**Figure 5:**
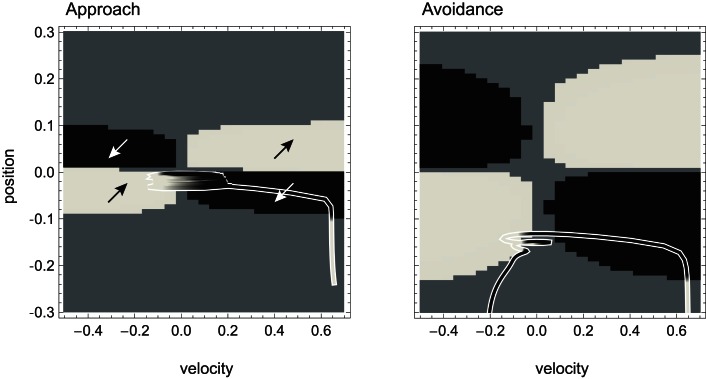
**Attractor landscape of evolved agent in approach and avoidance conditions**. Plotted here is the steady-state motor output as a function of fixed sensory input resulting from a given motor command (velocity) at a given position. Black attractor regions correspond to negative and white regions to positive steady-state velocities (approximately −0.2 and 0.6, respectively). Gray areas indicate bistable regimes. Actual trajectories are overlaid and color-coded according to the attractor the agent moves toward at any given time. Arrows indicate approximate tendencies of state change for the different attractor regions.

In Figure 5 we show the result of carrying out such analysis for our model agent, i.e., the qualitative behavior of the agent for different values of its sensory input. Here, we have determined for each fixed sensor output (which is given by the surface of the SM environment as a function of position and motor command), the steady state of all the agent’s variables, i.e., the state to which the agent would ultimately converge given enough time (its attractors). Of these states, we have plotted only the coordinate of the motor command.

Comparing with Figure 4, we can see that the agent’s internal structure transforms the four (smooth) peaks and troughs of the sensorimotor environment into four (discrete) regions of attraction. The white regions here correspond to positive velocity attractors, and the black ones to negative velocity attractors. Additionally, in some regions the system is bistable (gray). In this area, which attractor the system tends toward depends on the internal state at the given time.

What are the salient regularities of this attractor landscape? Firstly, it reflects the same symmetries as the sensorimotor environment above, one due to the environment and the other due to the agent’s motor capabilities. It should be noted that this is not by necessity. However, the requirements of the task here have led the agent (or rather the evolutionary algorithm) to preserve in its internal dynamics those features of the environment that are necessary for achieving the task.

Secondly, the attractor landscape implements a binary choice. Depending on whether the sensory values are above or below a certain value, the system will tend to move in either one or the other direction (indicated by the arrows in Figure 5). One prediction we can make based on this structure is that since there is no attractor that would lead to zero velocity, the only way the agent can stay close to a peak is by using changing sensory inputs to oscillate back and forth between different attractors. This is what we observe (Figures 4 and 5). Looking at the agent’s trajectories within this attractor landscape, one can also explain the difference between the approach and avoidance behaviors. Even though the possible steady-state motor outputs are identical in both types of environment, the regions in which they can be found differ in size (left and right plots in Figure 5). This results in the initial approach of the agent toward the shapes being identical. But when faced with wider shapes this trajectory does not carry the agent as far through the attractor region as it does for narrow shapes. Therefore, when the agent leaves the initial attractor region, it manages to enter into an asymmetric cycle of transitions between the two attractors in one case (approach), but gets fully captured by one of the attractors in the other (avoidance). The asymmetry of the oscillating approach pattern, in turn, can be explained by the difference in absolute steady-state velocity of the two attractors (approximately 0.6 and −0.2).

From a wider perspective we can therefore say that the agent’s attunement to the environment and task at hand depends on its internal structure. The attractor landscape transforms the SM environment into actions that conform to the achievement of the task. The attractor landscape, itself modulated by sensory perturbations, also determines which areas of the sensorimotor environment the agent will visit and how – in other words, what we have defined as the SM habitat. For every sensory perturbation this landscape provides information about which action the agent will take, and therefore what the following sensory stimulation will be as a consequence (one can in fact make this relation explicit, and express the SM habitat as the sensory consequence of letting the agent act on a given sensory perturbation for a certain amount of time, see Appendix).

#### Sensorimotor coordination

**Figure 6:**
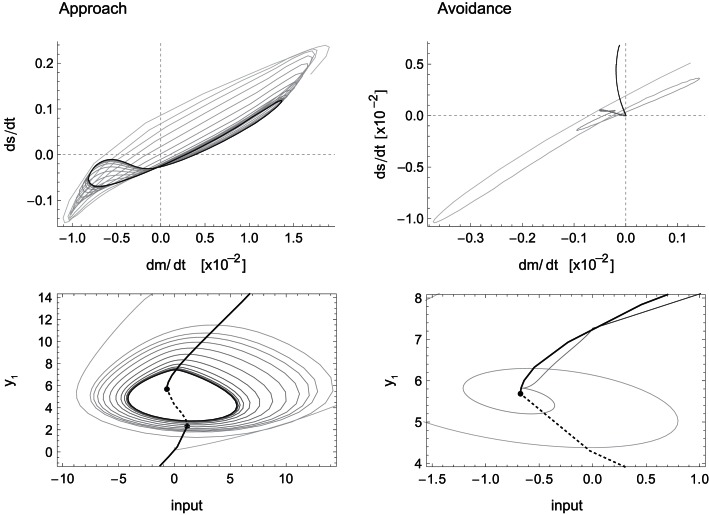
**Sensorimotor coordination patterns for approach condition (left) and avoidance (right)**. Top row: change in sensory activation (ds/dt) as a function of change in motor commands (dm/dt) during the agent’s interaction with the bell shape. After an initial transient, the system settles into a stable oscillation in the approach but not the avoidance condition. Bottom row: the same coordination patterns as trajectories in the hidden neuron’s bifurcation diagram, i.e., in coordinates of sensory input and the hidden neuron’s steady-state activation y_1_. The positions of the attractors, i.e., the states to which the system would settle in the long term, are indicated by solid lines emanating from the bifurcation points (filled circles). Note that in the case of approach, the system oscillates back and forth between the two attractors, while in the case of avoidance it gets eventually captured by one of them.

Finally, we show how SM coordination patterns contribute to task performance. Figure 6 (left) shows a convergence to a lower dimensional pattern in sensorimotor rates of change for the case of approaching behavior. This stable lower dimensional pattern is evidence of a mutual, closed-loop influence between sensory and motor variables. In other words, this is evidence that motor variables not only determine sensory patterns, but are themselves not independent variables. The fact is plainly visible and obvious in this model, but it remains conceptually crucial in wider discussions of the sensorimotor approach where often no mention is made of what determines the motor patterns that affect perception in the first place.

The particular SM coordination pattern also reinforces a functional relation to the environmental object, in this case, the behavior of staying close to the peak performing an oscillatory scanning motion. For avoidance (Figure 6 right) this is not the case, as no stable sensorimotor pattern in relation to the object is required, just its avoidance. However, according to our definition, and the fact that this transient behavior obtains reliably in the presence of wider peaks and that it results in a behavior that contributes to the desired performance, this also counts as a case of SM coordination.

For a pattern to count as SM coordination the convergence to a (meta)stable lower dimensional dynamics or its reliability in general is necessary but not sufficient. As illustrated in this case, it is also necessary for this pattern to be functional to the task. In our simple model, functionality is achieved by establishing an oscillatory pattern around the right hand side of the narrow peak and reliably *not* establishing any stable pattern when the gradient corresponds to the wider peak.

Another important aspect concerning SM coordination is illustrated in the bottom row of Figure 6. This is the fact that SM patterns do not correspond to, nor are they determined solely by, endogenous “brain” dynamics. As can be seen in the case of approach (left), the stable neural pattern cannot be trivially deduced from features in the agent’s internal state space (limit sets), or changes therein (bifurcations). Rather, what happens here is that the agent’s state is always “chasing” one attractor or the other, without ever being captured. Because the agent’s sensory input changes as a result of its movements (on the same time scale as the behavior), so does the asymptotic steady state that the agent tends to. In other words, the agent is always on a transient toward an attractor that is regularly changing its position. Interestingly though, the overall pattern that emerges forms a stable oscillation, which corresponds to a limit cycle of the coupled agent-environment system. In other words, the environment plays as much a part in the creation of the observed SM coordination pattern as the agent’s internal dynamics.

In summary, the different kinds of SMCs we have introduced contribute to a full explanation of the observed behavior. SM coordination patterns determine a relation between lower dimensional reliable trajectories in sensorimotor dynamics and their contributions to task functionality. The SM habitat establishes the “rules” that explain the presence of SM coordination by examining the dynamics of the coupled internal and environmental variables. And the SM environment establishes the wider constraints to the SM habitat such as symmetries that depend solely on the environmental and embodiment structures.

In addition to verifying the fact that the proposed kinds of SMCs are measurable, the minimal model also highlights some aspects of the SM structures that are not immediately obvious from their definitions alone. These include the observation that regularities in one SM structure can be preserved in another if this helps solving the task even though this need not generally be the case; the non-reducibility of SMCs to “brain” dynamics alone; the fact that SM coordination patterns serving different functions (approach and avoidance) can be implemented in a single, non-differentiated system (functional but no structural modularity); and the role of the environment as an equal player in the “selection” of which SMC to enact at a given time. The model brings home these subtle aspects of the definitions in a way that would have been difficult to arrive at otherwise.

The simplicity of the task we have chosen does not permit an easy illustration of the concept of SM strategy. The model could be modified to this effect, but the more complex analysis would be less effective for illustration purposes. A SM strategy entails the organization of various possible SM coordination patterns according to goals and norms. This requires a more complex scenario involving more shapes and more actions with a many-to-one viable or desirable relation. Wider norms (e.g., energy efficiency) or more general longer-term goals – both of which could be externally imposed, for example, by modifying the fitness function – would help determine what action to perform on a given object. We expect this process to happen at higher levels of dynamical modulation. Luckily several other models illustrate this possibility, and demonstrate a possible way forward in the modeling of norms and values as grounded in an agent’s self-maintenance (Di Paolo, 2000; Iizuka and Di Paolo, 2007; Barandiaran and Egbert, 2013). For theoretical discussions relating norms to enactive accounts of agency compatible with our use in this text also see (Di Paolo, 2005; Barandiaran and Moreno, 2008; Barandiaran et al., 2009; Di Paolo et al., 2010; Silberstein and Chemero, 2011).

Even though we have not explicitly illustrated the issue of SM strategies in our minimal model, this concept is no less important. Sensorimotor contingency theory relies heavily on notions such as mastery, skill, or attunement, which are inherently normative. The concept of SM strategies allows us to speak about how we move from SM coordinations to their organization, by providing a definition of what know-how at the sensorimotor level could mean.

## Discussion

The purpose of this text is the clarification of key concepts underlying the sensorimotor approach to cognition. As such, we make no claims as to whether or how this approach is intrinsically better than others. We only hope to have demonstrated that an operationalization of the concept of SMC can go some way in elucidating previously under-emphasized implications of the theory and can encourage progress in debates that might rest on conflicting interpretations of this concept.

A criticism that could be leveled against our dynamical definitions is that their practical application will be severely limited by the fact that we seldom know all the relevant equations that govern a cognitive system. This is indeed true if we were to demand a full dynamical account. Although formulated in the language of dynamical systems theory, the four definitions can be expressed using other methods, like information-theoretic measures or probabilistic approaches. For instance, in a robot study, Maye and Engel (2011) describe in probabilistic terms the transitions between sensed situations and action outcomes of the closed-loop system, constructing what can be considered slices through a stochastic SM habitat. Similarly, Schmidt et al. (2012) have studied the statistical information transfer between the different sensors and motors of a compliant quadruped robot that is controlled by externally induced (open-loop) leg actuations. Their approach reveals that directional correlations between sensors and motors depend on the environment (type of floor surface) and the body structure (hip actuators driving hip angle sensors in the same leg). The information extracted from this open-loop test thus corresponds to the SM environment in the dynamical account. Lungarella and Sporns (2006) also show that sensorimotor regularities induced by closed-loop agent-environment interaction can also be quantified using information theory. They observe that the structure of information flow in sensorimotor networks is temporally and spatially specific, dependent upon morphology, and modifiable with experience. While thus confirming that sensorimotor regularities are the result of constraints impinged at different levels, in this work the authors do not demonstrate how the overall informational structure observed results from the separate constraints of the environment, morphology, internal dynamics, and task demands. That this is possible in principle we have demonstrated by showing how task-specific SM coordinations are the result of regularities in the SM habitat, which itself is constrained by features in the SM environment.

Arguably, an interesting aspect of the sensorimotor approach is its agent-centredness, i.e., taking into consideration the agent’s embodiment, situatedness, skills, and goals. As suggested, the four kinds of SMCs can be arranged along a dimension from external to agent’s perspective of analysis. The SM environment requires for its definition the least amount of detail about the agent. It corresponds to all agents with similar sensors and effectors in a similar environment. The SM habitat is more agent-specific and adds the agent’s internal dynamics and closes the sensorimotor loop. The SM coordination patterns bring on a particular task-oriented dimension. And finally, the SM strategies add a normative dimension with the inclusion of value for the agent (efficiency, degree of skill, etc.).

In a similar vein, time plays different roles in each of the four kinds of SMCs. All of the structures can be time-dependent in the sense that external dependencies on time can alter both the environment and the agent (e.g., seasonal rhythms, or the effects of age and wear). Other than this, the SM environment is “atemporal”: it describes all possible sensory consequences of freely introducing a motor change. The SM habitat involves the set of possible trajectories. As such, it provides dynamical information and introduces notions such as trends, attractor landscapes, oscillations, etc. SM coordination patterns entail a more “local” element of temporality than that of dynamical trajectories because they rely on the fine-grained exercise of specific agent-environment engagements with the added constraint of contribution to functionality. Elements of duration, rhythms, etc., become crucial for this contribution; for instance, oscillatory patterns different from those observed in our simulated agent around the narrow peak might not contribute to the task. Finally, SM strategies add to this latter aspect that of a temporal organization among SM coordination patterns. Efficiency, resilience, or other normative evaluations will be affected by how patterns are coordinated in time, whether they run in parallel or in sequence, whether there are hard deadlines, delays, and so on.

The definitions also highlight the relevance of the determinants of action, a question that has been rather absent in the sensorimotor approach. Except for the SM environment, which describes a dependency of sensory activity on motor changes, the other kinds of SMCs are strictly speaking sensorimotor *co*-dependencies, as the loop is closed. Sensorimotor theory has been formulated in terms of SMCs, but, lacking a definition, the latter have been almost exclusively illustrated as one-way sensory dependencies on motor action (recall the line and retina example). Action has been treated more or less as a free variable in many of these illustrations. By this we mean that the appropriate action in a perceptual context is brought into an explanation of perception as required and without constraint. The squeezing movement of the fingers constitutes in part the softness of the sponge and the stroking movement of the hand constitutes the smoothness of the table surface. But what calls forth these particular movements in each case? Why don’t we stroke the sponge and squeeze the table? In each case what counts as appropriate action is in part also constituted by the perceptual context – i.e., action is perceptually constituted – and this aspect has been underdeveloped in sensorimotor theory. Except for SM environment, the other kinds of SMCs revert this situation at the most basic level by including closed-loop dynamics explicitly.

Putting the accent on this point is relevant for testing the implications of the approach for novel perceptual situations (including perceptual augmentation by means of sensorimotor prostheses). We come to a novel perceptual context for the first time already equipped with bodily know-how. We do not confront the new situation in a naïve manner but use our existing skills instead. There is not only a dependence of perception on motor activity but also a dependence of bodily movement on proprioception and the emerging perceptual awareness. This situation can converge into a stable way of exploring the perceptual situation and the development of this stability is what we could call the progressive mastery of SMCs.

A related point concerns the question of how the kinds of SMCs can help classify different forms of plasticity that are able to support the acquisition of novel skills, their progressive mastery, and their change over time. Plastic reconfigurations can occur at different levels and at once involve all the sensorimotor structures. However, we can draw some broad distinctions by asking for each sensorimotor structure, what would be the *minimal* change necessary for a plastic re-organization to occur (leaving the global structure of the more general kinds of SMCs intact). This helps us approximate a ‘hierarchy’ of plasticity going from minimal to more drastic reconfigurations.

A minimal plastic change in SM strategies would imply the re-organization of the relation between unchanged SM coordination patterns. This does not need to involve the acquisition of new SM coordinations, but simply an adjustment of the strategy with which they are used. In turn, minimal plastic changes in patterns of SM coordination imply only the fine-tuning of dynamical parameters so as to improve functionality, changing a skill, or learning a new task. Here, without any major re-organization of the regularities of the SM habitat, some changes, – e.g., changing the period of an oscillatory pattern such as those exemplified in our model – can count as sufficient for a adjusting a SM coordination pattern. Minimal plastic changes in SM habitat imply the re-organization of internal dynamics – e.g., generating novel possibilities for action and perception – without necessarily altering bodily structures (e.g., perceptual learning or re-habilitation). In contrast, plastic changes in the SM environment necessarily imply modifications to the body, the environment or both. Bodies change over time – growth, training, injury, incorporation of a prosthesis, aging – and the conditions of the environmental dynamics change as well. Such durable changes directly alter the most basic sensorimotor structure and will likely propagate to all the others.

The four kinds of SMCs can clarify the similarities and differences between the sensorimotor approach and ecological psychology (Gibson, 1979). According to the latter a much neglected constitutive factor of cognition is the structure of the environment: agents are thought to directly perceive the world by picking up invariants in the sensory array, i.e., properties that remain constant across transformations produced by self-motion (see e.g., Mossio and Taraborelli, 2008). This view thus conceives of a rich structure arising from the world and the properties of the body – in our terms, the SM environment – without which we could not explain the behavior of an agent. The sensorimotor approach acknowledges the importance of this structure. However, in the ecological approach, the origin of the particular motor patterns that bring about the invariant-revealing transformations is considered irrelevant. This implies that the SM environment is deemed exhaustive for the constitution of perception. We have seen, however, that it is actually insufficient. Key regularities are found in the closed-loop scenario in which the agent’s internal dynamics play a co-constitutive, irreducible role. These regularities are described by the SM habitat and, as we have shown, cannot be deduced from the SM environment alone. In this sense, the sensorimotor approach, in the interpretation afforded by our SMCs definitions, presents a more complete picture of perception, because it gives an account of motor-independent, open-loop sensory invariants as a special case, but explicitly acknowledges, in addition, the role of agent-specific, closed-loop sensorimotor invariants.

Can the four kinds of SMCs also throw light on some of the more general debates mentioned at the beginning regarding the wider claims about the constitutive role of SMCs know-how? We think that at least at this stage they can contribute by clarifying the terms of ongoing discussions such as the nature of the linkage between subpersonal and personal phenomena, i.e., between SMCs and perception. As we have mentioned, phrases like “mastery of the laws of SMCs” have been given various, sometimes conflicting, interpretations. A possible construal is that mastery consists in the acquisition of knowledge at the personal level (accessible to action planning) about the nature of subpersonal processes (SMCs). Now, some argue that the notion of knowledge in SMC theory should be abandoned, in favor of the idea that the enactment of SMCs suffices to account for the qualitative differences in perceptual experiences (Hutto, 2005). Others emphasize that perceptual experience in the absence of overt movement, or the perceptual presence of parts of object that are currently occluded from view, can only be explained by reference to the deployment of acquired knowledge (Roberts, 2009). What is at stake between these contrasting views (whether to understand mastery as enactment or as a form of knowledge) seems to be the nature of the know-how of sensorimotor regularities that are not actualized at a given time. How could these virtual regularities (e.g., the expectation that the rear part of a solid object is also solid were we to walk around it), inform, let alone constitute, our current perception?

While not fully resolving this issue, the use of the four kinds of SMCs in explaining the behavior of our evolved agent may throw some light on the matter. As our analysis has shown, the behavior of the agent cannot be predicted from the environment or the sensorimotor embodiment alone, but is the result of a coupling between internal processes and sensorimotor dynamics. This coupling is what transforms environmental and sensorimotor regularities into movement tendencies and their sensory consequences. One of the non-trivial insights provided by the model is that, crucially, there is nothing in the internal dynamics of the agent’s “brain” that represents the SMCs that are being enacted or the non-actualized sensorimotor regularities that still have a dynamical influence (see our analysis, e.g., Figure 5). Regularities in the dynamical landscape certainly exist and have a role in the production of behavior, but they are demonstrably not internally represented by the agent in any way. In particular, as should be clear from our analysis, regularities in sensorimotor structures are not determined by neural network parameters (e.g., connection weights), but rather co-determined by the agent’s internal structure, its sensorimotor interfaces, and environmental properties.

Another unexpected insight gained by the analysis in our model suggests that the question of direct access vs. represented knowledge may rely on false assumptions. These seem to be: (1) only what happens here-and-now can be accessed directly; and (2) anything that is accessed about what does not happen here-and-now, must somehow be “brought” into the here-and-now via representations (typically some internal register of the past or prediction about future states). The model shows that the first assumption is misleading and consequently, the second unnecessary. Dynamically, we should interpret the present sensorimotor state in terms of a “thick” here-and-now, that is, the current situation is not only the states that are actualized, but also the virtual traces and tendencies entailed by them.

As we have seen, strong tendencies defined in the SM habitat heavily constrain the actual trajectories of the agent (Figure 5). On the one hand, these tendencies underlie the separation of initially similar states according to their developmental history. The current state, in other words, reflects a history of changes that the system has undergone over time. In this way, the totality of past events is brought to bear on the current situation. On the other hand, these tendencies establish bounds on the set of possible future states. As long as the system does not suffer significant perturbations the current state entails to some extent the subsequent course of events, i.e., the flow of the system toward a specific subset of its state space.

This “temporal thickness” of dynamical systems has been linked explicitly with phenomenological investigations of the temporality of intentionality (van Gelder, 1999; Varela, 1999; Kelly, 2000). We can make a similar dynamical interpretation of Merleau-Ponty’s (1945/2012) concept of motor intentionality: in the acquisition of everyday skills the accumulation of experience serves to discriminate, with increasing specificity, situations that solicit a particular response (separation of traces). Experience also allows a person to incrementally refine her dispositions to respond to these solicitations (tuning of tendencies). Merleau-Ponty suggests that a response to a situation takes the form of movement toward the completion of a Gestalt (“maximum grip”) or equilibrium to which the body tends to relax without the need to mentally represent this optimum (like finding the right distance to admire a painting).

From this perspective, acquiring sensorimotor skills does not imply the use of representational knowledge to deal with non-actualized sensorimotor regularities. It is rather the shaping of dynamical tendencies that channel appropriate actions on the basis of past experience and in accordance with goals. Mastery, in this dynamical view, would be a measure of how, through the course of development and skill acquisition, an agent becomes increasingly sensitive to the entailed virtual traces and tendencies in the thick here-and-now.

## Conclusion

We propose four measurable sensorimotor structures that correspond to four different kinds of SMCs. Each of these structures presents various relevant regularities. These regularities, we suggest, are the “laws” or “rules” of SMCs that form the basis of the sensorimotor theory of perception. We have kept our definitions quite general and have not said much about important issues – such as introducing distinctions between modalities – to try, in the first instance, to establish the widest possible theoretical base. In spite of their generality, three of our SMCs definitions have been shown to be useful in explaining the behavior of a dynamical model of visually guided categorization. The fourth can easily be explored in extensions of this model.

Although alternative or new kinds of SMCs may be possible, we believe that at least these four have shown their potential, at once proving the points that a clear operational definition of SMCs can be provided, filling in a gap in the existing literature, and that the concept is not unitary, as might have been thought.

The dynamical definitions have also been useful for an initial examination of differences in aspects of temporality, agent involvement, and types of plasticity, as well as for drawing sharper theoretical demarcations between the sensorimotor and ecological perspectives on perception. Their roles in our model reveal that ideas such as mastery or know-how of SMCs allow dynamical interpretations that do not necessarily call forth more traditional representational stories, thus lending support to a more radically embodied understanding of the sensorimotor approach.

## Conflict of Interest Statement

The authors declare that the research was conducted in the absence of any commercial or financial relationships that could be construed as a potential conflict of interest.

## Figures and Tables

**Figure A1:**
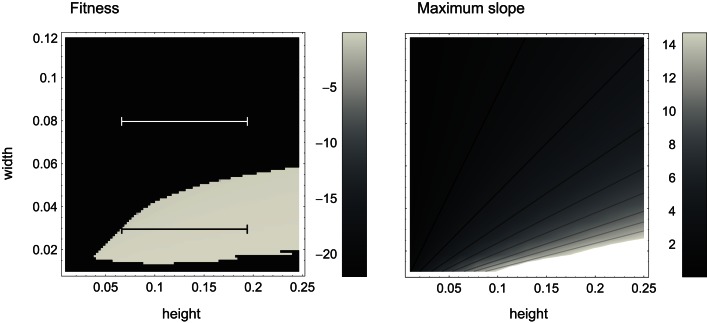
**Evolved performance**. Left: fitness of the evolved agent for approach behavior with single bell shapes of varying width and height. Higher (brighter) values are better, zero being the maximum fitness. Horizontal bars indicate the range of shapes encountered during evolution (widths = 0.03,0.08, height = 0.13 + −50%). Right: maximum slope of the bell shape (derivative of corresponding Gaussian at w) covering the same range of width and height. Black lines trace equally spaced contours of constant slope.

**Figure A2:**
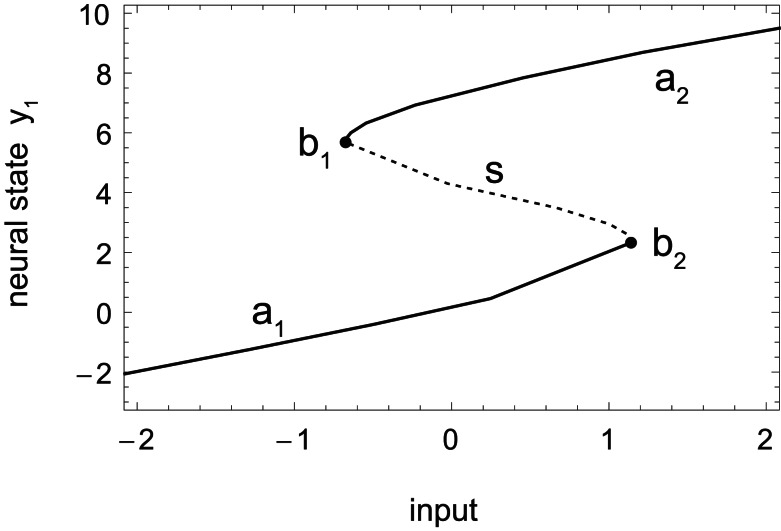
**Steady state of the hidden neuron a (y_1_) as a function of a fixed input**. For sensory stimulations smaller than b_1_ the output of the neuron evolves toward a single point attractor (a_1_), the position of which changes with the input value and is represented by a solid line). At the exact value of b_1_ a saddle-node bifurcation creates a new fixed point (a_2_) and a saddle node (s, dashed line). As input increases further, both fixed-points move toward higher levels of hidden neuron activation. Eventually, in a second (reverse) saddle-node bifurcation at b_2_, fixed-point a_1_ collides with the saddle node and both vanish, leaving only attractor a_2_.

**Figure A3:**
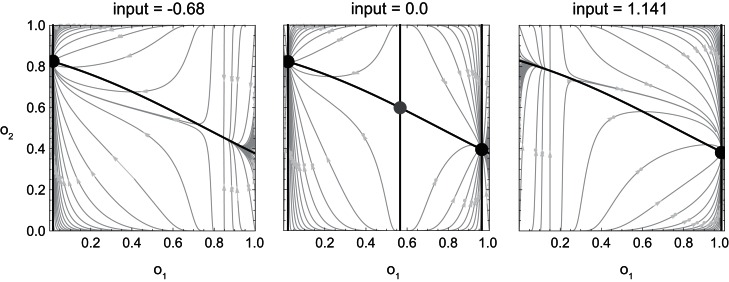
**Phase portraits surrounding the bifurcation points in coordinates of neural output**. Variable o_1_ corresponds to the output of hidden neuron a, and o_2_ to motor neuron m. Black lines show isoclines, at the intersection of which can be found the fixed-point attractors (black dots) and saddle node (gray dots). In light gray the flow of trajectories toward the fixed points is shown.

**Figure A4:**
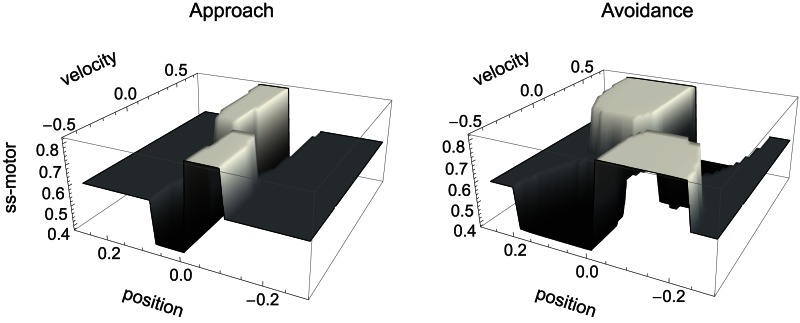
**Attractor landscape of evolved agent in approach and avoidance conditions**. Steady-state motor output as a function of fixed sensory input resulting from a motor command (velocity) at a given position. Black attractor regions correspond to negative and white regions to positive steady-state velocities. Gray areas indicate bistable regimes.

**Figure A5:**
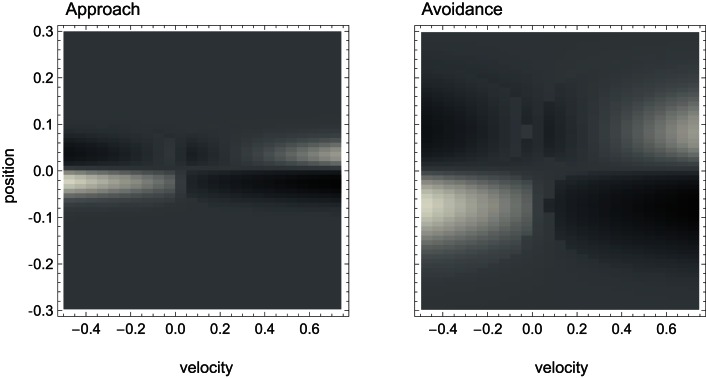
**Sensorimotor habitat**. Change in sensory reading as agent moves a small proportion from its initial position and velocity toward its steady-state velocity. Bright areas indicate increased, dark areas reduced sensor values.

## Footnotes

^1^The formalization corresponds to an abstract case. Specific details depend on the case under consideration: the equations may or may not depend explicitly on time and they may exhibit different degrees of non-linearity, piecewise, or conditional functional relations, and dependence on various parameters. Equations are valid within a timeframe. In a system capable of plastic adaptation, some functions will change over time. It is possible to formalize some of these changes through the inclusion of rules of plasticity. Other changes are harder to capture, such as the emergence of new relevant variables through development or during the acquisition of novel perceptual systems. We want to emphasize that this general formal description is not necessarily complete, but it is sufficient for our current purposes, and as we shall see, once introduced, the new kinds of SMCs can be investigated by a range of other methods that do not demand the level of detail of a full dynamical description.

## Appendix

### Details of agent and environment

The model agent introduced in Section “A Minimal Model: Categorical Perception” can move along a one-dimensional and wrap-around environment (see Figure 1). The environment extends over 1 (arbitrary) unit in space, and the agent’s maximum velocity is 1 unit per second. Within this environment, the agent is presented with visual stimuli in the form of two bell-shaped curves of different widths *w* (0.03 and 0.08), defined by the following Gaussian equation:

$$E\left(p\right)=h\cdot e\frac{{\left(p-x\right)}^{2}}{2w^{2}}$$

here *h* is the height of the shape, *x* the position of its peak, and ±*w* corresponds to the maxima of the function’s derivative, i.e., to the inflection points where its slope is steepest. Values of the Gaussian function are truncated to 0 beyond —*x*— > 10*w* for reasons of computational efficiency. From trial to trial the two shapes are presented in random horizontal positions while observing a minimum guaranteed separation. They also vary in height (0.13 ± 50%), but such that within each trial their height is equal.

The sensor *S* measures the proximity of the Gaussian shape to the agent’s position (shortest distance, i.e., in the direction directly ahead of the agent):

$$s=S\left(e\right)=1-\frac{d_{\max}-e\left(p\right)}{d_{\max}},$$

where *d*_max_ is the maximum distance between the agent and the shapes (where the Gaussian approaches 0. Sensor values therefore range from 0 where the shape is flat to 1 where the vertical distance between the shape and the agent is zero.

The controller of the agent consists of a small (2-node) continuous-time, recurrent neural network (CTRNN). Each node in this network is governed by the equation

$$\tau_{i}{ẏ}_{i}=-y_{i}+\sum_{j=1}^{n}w_{ji}\;\sigma \left(y_{j}+\theta_{j}\right)$$

where *y*_i_ is the activation of node *i*, τ_i_ its time constant, *w*_ji_ the strength of the connection from node *j* to *i*, θ*_j_* a bias term, and σ*_j_* the logistic activation function. One of the nodes (a in Figure 1) receives as input the time-derivative of sensor reading *s* as an additional term in the equation. The other node (m) delivers continuous motor commands. Its output is remapped to the range [−1, 1] and specifies directly the velocity of the agent (to the left or to the right depending on the sign).

In terms of the general variables introduced in Section “Four Kinds of SMCs,” environmental states **e** are described in this simulation model by the Gaussian functions describing the two shapes: **e** = *E*(**p**), where the body configuration **p** is simply the agent’s horizontal position. The sensor S transforms environmental variables **e** into sensory state **s** = S(**e**), the time-derivative of which serves as input to the neural network. The agent’s internal state **a** corresponds to the vector representing the activity of the neural network’s hidden node (a in Figure 2) and motor neuron. The latter ultimately drives motor commands **m**, which here correspond to the sigmoided output of the neuron (m in Figure 2) that specifies the agent’s velocity.

The parameters of the agent’s neural controller are evolved using a microbial genetic algorithm (Harvey, 2011), with mutation as a random offset vector in the unit hypersphere (vector length chosen from a Gaussian distribution, and direction from a uniform distribution). The fitness function measures the root-mean-square error between the position of the agent and the narrow shape’s peak, averaged over the last two seconds of each 10 s trial. An individual’s overall fitness is then averaged again over 100 trials of random shape presentations.

Figure A1 (left panel) shows the ability of the best evolved agent for generalized discrimination of shapes over a range of widths and heights. The figure plots the agent’s fitness, i.e., the averaged distance from the peak, when presented with only one shape at a time. Brighter shades indicate close proximity and darker shades large distances. For the purpose of generating this picture only, we changed the environment from circular to open-ended. In this case avoidance of a shape always leads to the agent moving far away from the shape, preventing cases of returning to the shape after the first avoidance.

The figure indicates that the agent carves out a specific region of widths and heights for which it perfectly approaches the corresponding shapes, while avoiding all others. Which is to say, from a continuum of possible shapes the agent behaviorally forms two distinct categories. The shapes encountered during evolution are shown as horizontal lines, and clearly fall into the two distinct regions. Widths are not discriminated independently from height however. As shape height increases, progressively wider shapes fall into the “approach” category. Since the task was designed such that shapes could only the distinguished by their steepness (but not their absolute values), we show in the right panel the maximum slope of the bell shape (i.e., the derivative of the Gaussian function at point *w*) for the same range of widths and heights. The boundary between the two categories seems to be related to the steepness of the shape, but shows a non-linear falloff at greater heights.

### Dynamical analysis

In the dynamical analysis of the model agent’s SM habitat we followed a two-step process determining:

1. The effect of sensory stimulation (as mapped out completely by the SM environment for all possible agent positions and motor commands – see Figure 3) on the steady-state of the agent’s dynamics. Here we fix sensory stimulation at each possible value and identify the limit sets of the agent. The resulting attractor landscape (Figure 5) can be seen as describing qualitatively all trajectories that can be reached by the agent for fixed sensory values.
2. The effect of the identified attractor landscape on the subsequent change in sensory values.

This provides us with information about the SM habitat in the form of (1) the effect of sensory changes on motor commands, taking into account internal dynamics and (2) the effect of internally driven motors on subsequent sensor values. Together they describe the whole loop, while taking into account the internal dynamics. However, since this method only considers the system’s steady states for fixed input values (which in the behaving agent would normally be time-varying), it misses information about the transient behavior of the system.

Starting with the qualitative description of the agent’s dynamics as a function of its input, analysis reveals that the hidden neuron exhibits bistable dynamics (see Figure A2), while the motor neuron performs a simple rescaling of the hidden neuron’s activity. As input to the hidden neuron increases from negative to positive values, the neuron undergoes two bifurcations (at input levels of b1 ≈ 0.67 and b2 ≈ 1.14). This leads to three types of qualitatively different dynamics characterized by the appearance and destruction of a saddle-node separating two fixed-point attractors in the mid-range of input levels.

In Figure A3 we show the two-neuron system’s phase portrait at input levels surrounding the bifurcation points (using neural output coordinates). The salient point here is that the system will always tend to one of two fixed points. The first is located around motor neuron levels of approximately 0.8, and the second around levels of 0.4. Translated into agent velocities this implies that the agent will always tend, in the steady state, to either a positive velocity of 0.6 or a negative velocity of −0.2 (note the asymmetry). In the bistable regime, which of the two attractor basins the system finds itself in, is determined by the activity of the hidden neuron. All states smaller than the saddle-node will tend to the positive velocity attractor, while states on the other side of the node will move toward the negative velocity attractor.

Next, we can show directly how these attractors (the long term output of the neural activity for a given fixed sensory input) relate to the SM environment illustrated in Figures 3 and 4. To do so, we determine for all possible sensory values (given by the surface in Figure 3 for each position and velocity) the resulting attractors of the autonomous agent system (this method has been already applied for the dynamical analysis of minimally cognitive agents by Beer, 2003). Of the attractor’s coordinates we plot only the equilibrium value of the motor neuron, as this directly affects the agent’s behavior. The resulting surfaces for approach and avoidance are shown in Figure A4.

Figure 5 in the main text was derived from these surfaces by adopting a top-view and by adding the agent’s trajectories color-coded according to the attractor the agent is tending to at each point in time (the surfaces in the figure appear pixelated because the equilibria take time to process and were hence calculated only at a certain sampling of the region).

As mentioned in Section “A Minimal Model: Categorical Perception,” we can describe the SM habitat also in terms of the sensory consequences that result from the agent acting on initial sensory perturbations for a certain period of time. A precise method would simulate the agent starting at all possible pairs of position and velocity and compare how sensory values change after a given amount of time. Here we use an approximation only. First, we calculate the sensory stimulation for all combinations of initial position and commanded velocity *s_0_* = *s(p_0_, v_0_)*. Next, we identify the steady-state velocity *v_s_* for (constant) sensory state *s_0_* as given by the attractor landscape. We then assume that the system relaxes toward the attractor, and hence that the agent’s velocity will change a small proportion from *v_0_* toward *v_s_: dv* = *(v_s_* − *v_0_)/k*. Given this change in velocity we can calculate the new sensor reading *s_1_* at the new position *[p_0_* + *v_0_?dt, v_0_* + *dv]*, and finally calculate the change in sensor reading *s_1_* − *s_0_*. The result is a single diagram that summarizes, in an approximate fashion, the functional relation between sensors and motor commands while taking into account the agent’s internal dynamics. The procedure is informative for a sufficiently small finite change, and parameters were chosen in accordance with the timescales of the system (in this case, *k* = 0.5). For the two environmental conditions this is shown in Figure A5.

One can see that for areas of initially negative sensory stimulation (see e.g., *p* = −0.05, *v* = −0.5 in SM environment above, Figure 4), the tendency is to move toward more positive positions (see corresponding area in attractor landscape), and hence more positive sensory stimulation (i.e., positive change). For positive sensory stimulation, the tendency is to move toward more negative values (negative change). In fact, the surface resembles a negative image of the SM environment. One can interpret this surface as implementing the SMC rule “if the sensor currently reports a positive value, move such as to make it more negative, and vice versa.” Since sensor readings are also proportional to the slope of the bell shape, this rule explains why the agent should come to oscillate back and forth in an area where this change is small.
